# Education and Mortality in the Rome Longitudinal Study

**DOI:** 10.1371/journal.pone.0137576

**Published:** 2015-09-16

**Authors:** Laura Cacciani, Anna Maria Bargagli, Giulia Cesaroni, Francesco Forastiere, Nera Agabiti, Marina Davoli

**Affiliations:** Department of Epidemiology, Lazio Regional Health Service, Rome, Italy; University of Westminster, UNITED KINGDOM

## Abstract

**Background:**

A large body of evidence supports an inverse association between socioeconomic status and mortality. We analysed data from a large cohort of residents in Rome followed-up between 2001 and 2012 to assess the relationship between individual education and mortality. We distinguished five causes of death and investigated the role of age, gender, and birthplace.

**Methods:**

From the Municipal Register we enrolled residents of Rome on October 21^st^ 2001 and collected information on educational level attained from the 2001 Census. We selected Italian citizens aged 30–74 years and followed-up their vital status until 2012 (n = 1,283,767), identifying the cause of death from the Regional Mortality Registry. We calculated hazard ratios (HRs) for overall and cause-specific mortality in relation to education. We used age, gender, and birthplace for adjusted or stratified analyses. We used the inverse probability weighting approach to account for right censoring due to emigration.

**Results:**

We observed an inverse association between education (none vs. post-secondary+ level) and overall mortality (HRs(95%CIs): 2.1(1.98–2.17), males; 1.5(1.46–1.59), females) varying according to demographic characteristics. Cause-specific analysis also indicated an inverse association with education, in particular for respiratory, digestive or circulatory system related-mortality, and the youngest people seemed to be more vulnerable to low education.

**Conclusion:**

Our results confirm the inverse association between education and overall or cause-specific mortality and show differentials particularly marked among young people compared to the elderly. The findings provide further evidence from the Mediterranean area, and may contribute to national and cross-country comparisons in Europe to understand the mechanisms generating socioeconomic differentials especially during the current recession period.

## Introduction

The association between socioeconomic position (SEP) and health in high income countries has been widely studied and reported [1, 2]. SEP, in terms of its different individual or contextual component dimensions, is inversely and consistently associated with several health outcomes [3] in different health areas [4, 5]; moreover, socioeconomic health inequalities persist independently of the presence of highly developed welfare systems [6].

Well after the publication of the Black Report in 1980 [7], which demonstrated the widespread existence of socioeconomic differentials on mortality, about a decade ago social epidemiologists from different parts of Europe were still indicating the persistency and widening of the association between socioeconomic inequalities and mortality in Western European countries [8]. However, it was expected that the economic crisis of the recent years would have posed the conditions for an exacerbation of such differentials in an unpredictable way. A longitudinal Swedish research found that mortality following unemployment at older ages in men was associated with greater risk than at younger ages, with the greatest relative increase in risk among men with markers of better health [9], thus excluding the health selection hypothesis. More recently, a cross-sectional study on differential in mortality in 16 European cities (including Italy) reported an increasing pattern of poor health with socioeconomic deprivation based on occupation, educational level, and being foreigner from low income countries [10].

Mackenbach and colleagues suggested that the political history of Europe and different implementation of health policies are in part responsible for unequal health and rising of health inequalities among Europeans [11]. In addition, circumstances related to access to immaterial resources and changes in intergenerational mobility may in part explain the persistence of health inequalities in modern welfare states [6].

Among the indicators available to measure SEP at the individual level, education is often used in epidemiological studies [12, 13] and to explain health status [14]. It owns interesting peculiar characteristics: once completed, it is invariable over time; it may reflect socioeconomic circumstances in early life; it may be considered a good predictor of health of individuals in their later adulthood as well as in their offspring, as more educated people get better employment opportunities and higher income, and their knowledge and awareness about health and preventive strategies is higher compared to less educated people.

Although in Italy different studies evaluated the association between SEP and health, few of these investigations employed a longitudinal population-based approach using educational level, and recently none focused on residents of a large city. A study of temporal trends of socioeconomic differentials in premature mortality in Rome was based on administrative health data, and showed the persistency of inequalities at the beginning of 21^st^ century [15]. The role of both individual and contextual SEP on mortality was reported in Turin in a study analysing data from three census cohorts of residents in 1971, 1981, and 1991, showing a weaker decrease in mortality among the most disadvantaged groups [16]. Another study conducted on all Italian provinces showed a positive ecological association between income inequalities and mortality, in particular in low income geographical areas, with old women living in the south of the country being particularly vulnerable to the effect of income deprivation [17].

For the aim of the present study, we used the cohort of the resident population in Rome, Italy (the Rome Longitudinal Study, RoLS) [18], which includes population registry and census information, in order to evaluate the association between educational level attained and overall or cause-specific mortality between 2001 and 2012. The RoLS participates in the network of Metropolitan Longitudinal Studies that includes population based cohorts of residents in different Italian cities which represent acknowledged sources of data to study inequalities in health [19].

## Material and Methods

### Ethics statements

The RoLS is part of the National Statistical Program for the years 2011–2013 and was approved by the Italian Data Protection Authority. Consent was not necessary because we used data already collected at the beginning of the study and the data were analyzed anonymously; individuals cannot be identified directly or through identifiers and results are shown in aggregate form.

### Study design and data

**Fig 1:**
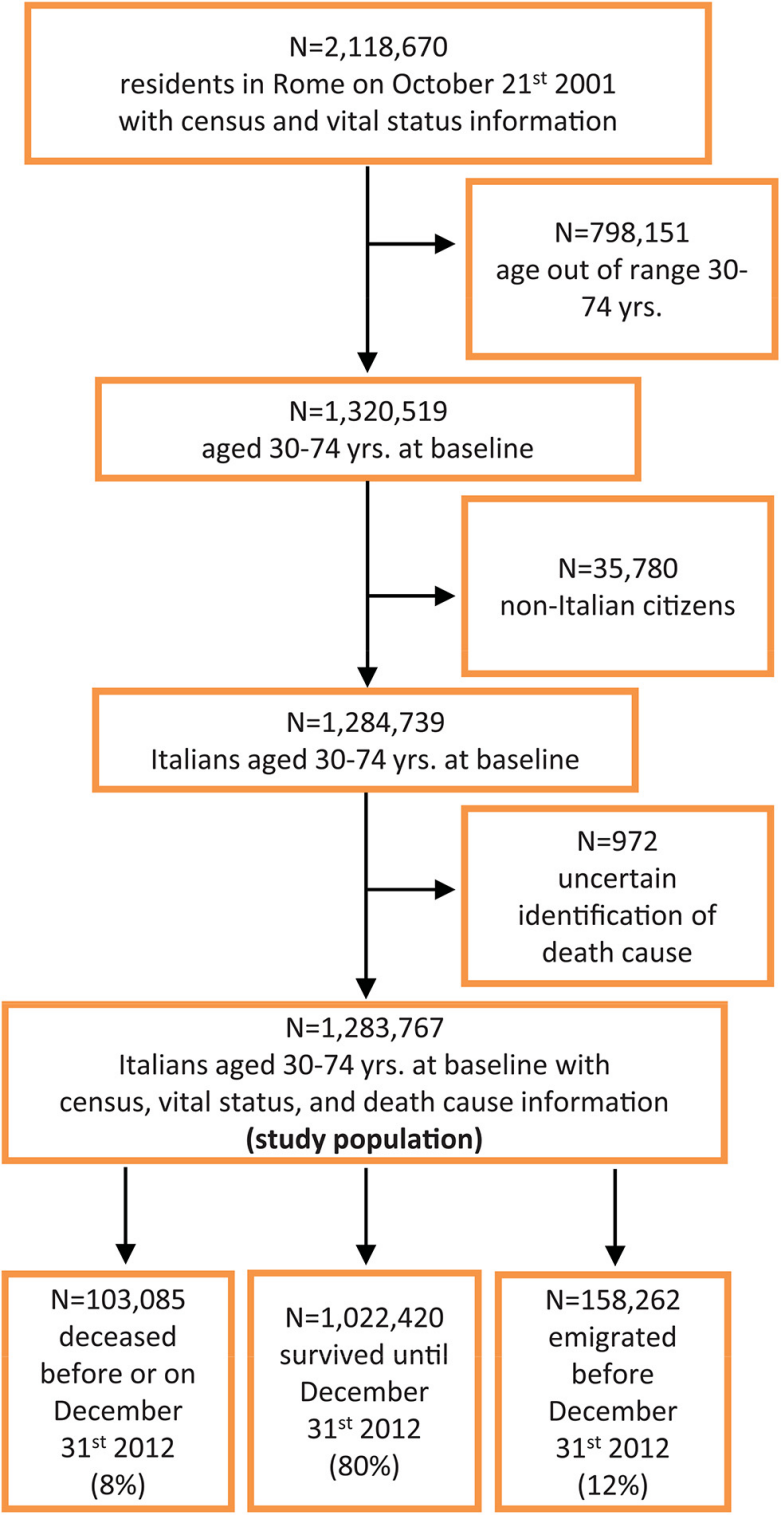
Flow-chart of the selection of the study population.

People residing in Rome, Italy (about 2.5 million inhabitants at the 2001 Italian census, National Institute of Statistics), were enrolled using data of the Rome Municipal Register on October 21^st^ 2001 and, through record-linkage procedures under strict control to protect individual privacy, information on the educational level attained reported in the 2001 Census of the Italian population was collected for 2,118,670 individuals [18]. We included only 30–74 year old Italians (n = 1,284,739) in order to have a complete and comparable history of education and to avoid selection bias potentially introduced by the immigrant status. Then we followed-up the vital status of the eligible population until December 31^st^ 2012, without allowing for future entries, and we linked information on death causes from the Regional Mortality Registry, excluding 972 individuals due to uncertain identification of their death cause. We finally analysed 1,283,767 individuals (99.9% of the eligible population); of these, 158,262 were lost to follow-up due to emigration during the study period (12%). Fig 1 shows the flow-chart for the selection of the study population.

#### Exposure, risk factors and outcomes

The exposure was the educational level attained at inclusion (October 21^st^ 2001), classified according to the International Standard Classification of Education (ISCED): no education (0), primary level (1), lower secondary (2), upper secondary (3), post-secondary or more (4, 5, 6, reference category). Gender, age, and birthplace were used as additional demographic risk factors in the context of this study.

The outcomes of the study were overall mortality (codes of the International Classification of Diseases, 9th Revision: 001–999) at the end of 2012, and mortality for the following specific causes: malignant neoplasms (140–208), diseases of the circulatory system (390–459), diseases of the respiratory system (460–519), diseases of the digestive system (520–579), injury and poisoning (800–999).

### Data analysis

We used Cox’s proportional hazard regression to evaluate the association between educational level and the occurrence of overall or cause specific mortality during the study period; current age in years was used as the main time axis [20]. Models with and without interaction terms for age groups at inclusion (30–44, 45–59, 60–74), gender, and birthplace (Rome or elsewhere) with educational level where then performed, and the Likelihood Ratio Test (LRT) was used to assess whether the interactions were statistically significant. We tested age and gender because educational level may have different effects according to these risk factors in a life course approach [21, 22], and we tested the birthplace as a proxy of early-life environmental exposures. In addition, we split the follow-up period into two time intervals, each holding equal numbers of deaths, to produce stratified Cox’s regression, thus accounting for time-varying relationships. We checked the proportional hazard assumption (PHA) by visual inspection of the log-minus-log survival plot. Models with an interaction term between time and education were performed. We considered two-sided p-values less than 0.05 as statistically significant. We used software Stata/SE 12.0 to perform statistical analyses.

#### Additional analysis: censoring due to emigration

Under the hypothesis of no unmeasured confounding and absence of informative censoring, the inverse probability weighting (IPW) approach was applied to handle loss to follow-up due to emigration [23, 24, 25]. A logistic regression model was specified to estimate the probability of non emigration (P), and then the inverse of the estimates were used as weights (W) in regression models evaluating the association between education and overall mortality. We used the following census variables to predict P: educational level attained, gender, birthplace (see above for categories), age, marital status (unmarried, married, divorced, widow), unemployment (yes/no). In order to calculated stabilized weights (SW), we also run a model including only the educational level (exposure variable) and then we re-estimated predicted stabilized probabilities (SP); SWs were calculated as the ratio between SP and P and used to estimate stabilized weighted hazard ratios (HRs) which were compared to weighted HRs.

This approach was used only in the analysis of overall mortality.

## Results

During the study period a total of 103,085 deaths were observed in the cohort. Table 1 shows the distribution of baseline characteristics of the study population and mortality rates. More than a half of the study populations attained an upper secondary degree, 53.1% were females, 37.3% were aged 30–44 years, and 55.3% were born in Rome. Among people aged 30–74 years, the overall crude mortality rate was 7.9 per 1,000 person-years (PYs) at inclusion. The highest crude mortality rate for each risk factor was observed among individuals without any education (21.6 per 1000 PYs), males (9.9), 60–74 year-olds (20.6), and among those not born in Rome (9.8). The highest cause-specific mortality rate was found for malignant neoplasms (3.6 per 1000 PYs).

**Table 1:** Distribution of baseline characteristics, and crude overall and cause-specific mortality rates of residents in Rome, 2001–2012.

Baseline characteristics	N (total = 1283767)	Column %	Deaths (total = 103085)	PYs (total = 12975217)	Mortality rates (per 1000 PYs)
Education					
None	34377	2.7	7147	331331	21.6
Primary	229618	17.9	35957	2259800	15.9
Lower secondary	342801	26.7	26782	3449425	7.8
Upper secondary	445270	34.7	21496	4552656	4.7
Post-secondary+	231701	18.1	11703	2382005	4.9
Gender					
Male	602021	46.9	59272	5976663	9.9
Female	681746	53.1	43813	6998555	6.3
Age group (yrs.)					
30–44	478786	37.3	6043	4941133	1.2
45–59	423421	33.0	20694	4330414	4.8
60–74	381560	29.7	76348	3703671	20.6
Birthplace					
Rome	710026	55.3	46795	7251703	6.5
Elsewhere	573741	44.7	56290	5723514	9.8
Cause of death					
All causes			103085		7.9
*Malignant neoplasms*			*46771*		*3*.*6*
*Circulatory system*			*30021*		*2*.*3*
*Respiratory system*			*4356*		*0*.*3*
*Digestive system*			*4025*		*0*.*3*
*Injury and poisoning*			*3629*		*0*.*3*
*Other causes*	* *	* *	*14283*	* *	*1*.*1*

S1 Table shows the frequency distribution of the study population by educational level stratified by age group at inclusion, gender, and birthplace; S2 and S3 Tables show the frequency distribution of the study population by educational level stratified by age group at inclusion and cause of death for males and females.

### Results from regression analysis

#### Overall mortality

Results from Cox’s regression suggested an adverse effect of low education on overall mortality of residents in Rome, decreasing with increasing level of education (ref: post-secondary+; adjusted for birthplace), HRs (95%CIs): 2.1 (1.98–2.17) for no education, 1.6 (1.56–1.64) for primary level, 1.4 (1.40–1.48) for lower secondary, and 1.2 (1.13–1.20) for upper secondary, among males; the estimates among females were 1.5 (1.46–1.59), 1.3 (1.21–1.30), 1.2 (1.15–1.24), and 1.1 (1.03–1.11) respectively.

We found statistically significant interactions when we run the regression model including interaction terms between the exposure with age, gender, or birthplace (LRT p<0.001); therefore, we reported stratified analysis for the risk factors considered (Table 2): low educational level showed a statistically significant inverse association with overall mortality in all strata; the strongest association was observed for young women born in Rome without education compared to those with a post-secondary+ level (HR 7.30, 95%CIs 5.29–10.08). The strength of association varied across strata of age, gender, and birthplace, and was lower among the elderly, among females, and among those not born in Rome.

**Table 2:** Results from the Cox’s regression model: HRs of the association between education and mortality of residents in Rome stratified by age group at inclusion, gender, and birthplace, 2001–2012.

Education (ref: post-secondary+)	Born in Rome								Born elsewhere							
	Males				Females				Males				Females			
	HRs	95% CIs			HRs	95% CIs			HRs	95% CIs			HRs	95% CIs		
	*All ages (30–74 yrs*.*)*															
None	2.60	2.42	-	2.80	1.96	1.81	-	2.11	1.81	1.71	-	1.92	1.38	1.30	-	1.46
Primary	1.84	1.77	-	1.92	1.38	1.30	-	1.45	1.45	1.40	-	1.50	1.17	1.11	-	1.22
Lower secondary	1.58	1.52	-	1.65	1.23	1.17	-	1.30	1.34	1.29	-	1.39	1.18	1.12	-	1.24
Upper secondary	1.21	1.16	-	1.26	1.06	1.00	-	1.12	1.15	1.11	-	1.20	1.10	1.05	-	1.16
	*30–44 yrs*.															
None	6.27	4.68	-	8.41	7.30	5.29	-	10.08	3.98	2.33	-	6.77	5.06	2.97	-	8.63
Primary	5.73	4.80	-	6.83	2.52	2.01	-	3.17	3.03	2.24	-	4.11	1.75	1.23	-	2.48
Lower secondary	2.70	2.37	-	3.07	1.77	1.53	-	2.05	2.36	1.94	-	2.87	1.66	1.33	-	2.08
Upper secondary	1.34	1.17	-	1.52	1.23	1.07	-	1.41	1.40	1.14	-	1.71	1.36	1.10	-	1.67
	*45–59 yrs*.															
None	3.47	2.84	-	4.23	3.68	3.05	-	4.45	3.12	2.61	-	3.72	1.64	1.38	-	1.95
Primary	2.15	1.97	-	2.35	1.51	1.36	-	1.68	1.95	1.79	-	2.13	1.24	1.12	-	1.37
Lower secondary	1.70	1.57	-	1.84	1.30	1.18	-	1.43	1.58	1.45	-	1.71	1.18	1.07	-	1.30
Upper secondary	1.30	1.20	-	1.40	1.13	1.02	-	1.24	1.28	1.18	-	1.39	1.09	0.98	-	1.20
	*60–74 yrs*.															
None	2.23	2.06	-	2.43	1.60	1.46	-	1.75	1.66	1.56	-	1.76	1.32	1.24	-	1.41
Primary	1.62	1.54	-	1.70	1.22	1.14	-	1.30	1.34	1.29	-	1.39	1.13	1.07	-	1.19
Lower secondary	1.38	1.31	-	1.45	1.09	1.01	-	1.17	1.24	1.19	-	1.30	1.14	1.07	-	1.21
Upper secondary	1.19	1.13	-	1.26	0.98	0.91	-	1.05	1.12	1.07	-	1.17	1.09	1.02	-	1.16

#### Cause-specific mortality

The regression analysis adjusted for birthplace suggested a decreasing inverse association of education with mortality among both genders for all causes of death (see Tables 3 and 4). The following HRs of no education vs. post-secondary+ level, respectively among males and females, were observed: malignant neoplasm 1.84 and 1.13; circulatory system 2.04 and 1.85; respiratory system 3.06 and 1.73; digestive system 3.39 and 1.98; injury and poisoning 2.28 and 1.52. When we looked at estimates stratified also by age group at inclusion, we found exacerbated inverse patterns between all specific causes of death considered and education compared to unstratified estimates, in particular for young people.

**Table 3:** Results from the Cox’s regression model: HRs of the association between education and cause specific mortality of residents in Rome, stratified by age group at inclusion and adjusted for birthplace. Males, 2001–2012.

Education (ref: post-secondary+)	Malignant neoplasms				Circulatory system				Respiratory system				Digestive system				Injury and poisoning				Total			
	HRs	95% CIs			HRs	95% CIs			HRs	95% CIs			HRs	95% CIs			HRs	95% CIs			HRs	95% CIs		
	*All ages (30–74 yrs*.*)*																							
None	1.84	1.72	-	1.97	2.04	1.89	-	2.21	3.06	2.54	-	3.68	3.39	2.74	-	4.19	2.28	1.78	-	2.92	2.07	1.98	-	2.17
Primary	1.57	1.51	-	1.63	1.57	1.49	-	1.64	1.88	1.65	-	2.15	2.05	1.78	-	2.37	1.73	1.50	-	1.99	1.60	1.56	-	1.64
Lower sec.	1.38	1.33	-	1.44	1.46	1.39	-	1.53	1.53	1.34	-	1.76	1.82	1.58	-	2.10	1.52	1.33	-	1.73	1.44	1.40	-	1.48
Upper sec.	1.19	1.14	-	1.24	1.18	1.12	-	1.24	1.03	0.88	-	1.20	1.20	1.03	-	1.40	1.14	1.00	-	1.31	1.17	1.13	-	1.20
	*30–44 yrs*.																							
None	2.57	1.43	-	4.62	6.97	4.26	-	11.41	35.53^§^	7.16	-	176.17	12.40	3.95	-	38.95	1.67	0.61	-	4.55	5.56	4.30	-	7.18
Primary	2.06	1.49	-	2.85	4.81	3.50	-	6.62	15.09	3.77	-	60.42	16.11	7.92	-	32.76	3.73	2.57	-	5.40	4.82	4.14	-	5.60
Lower sec.	2.00	1.67	-	2.40	3.02	2.41	-	3.78	6.19	1.88	-	20.41	6.96	3.74	-	12.94	2.11	1.66	-	2.69	2.59	2.33	-	2.89
Upper sec.	1.33	1.11	-	1.59	1.53	1.21	-	1.92	1.45	0.40	-	5.28	2.68	1.41	-	5.08	1.14	0.89	-	1.45	1.34	1.20	-	1.49
	*45–59 yrs*.																							
None	2.50	2.04	-	3.08	2.88	2.20	-	3.77	11.24	6.35	-	19.88	9.37	5.51	-	15.93	2.53	1.22	-	5.24	3.28	2.88	-	3.75
Primary	1.94	1.78	-	2.11	2.03	1.81	-	2.29	2.92	1.95	-	4.36	3.80	2.72	-	5.30	2.08	1.54	-	2.80	2.05	1.93	-	2.18
Lower sec.	1.53	1.42	-	1.66	1.75	1.57	-	1.95	1.75	1.18	-	2.59	2.66	1.94	-	3.66	1.59	1.21	-	2.09	1.64	1.55	-	1.74
Lower sec.	1.28	1.18	-	1.38	1.24	1.11	-	1.39	1.19	0.79	-	1.79	1.87	1.35	-	2.58	1.27	0.96	-	1.66	1.29	1.21	-	1.36
	*60–74 yrs*.																							
None	1.71	1.58	-	1.84	1.84	1.69	-	2.01	2.65	2.18	-	3.23	2.38	1.89	-	3.00	1.99	1.50	-	2.65	1.83	1.75	-	1.92
Primary	1.46	1.39	-	1.53	1.42	1.35	-	1.50	1.74	1.52	-	2.00	1.48	1.26	-	1.73	1.38	1.14	-	1.66	1.44	1.40	-	1.48
Lower sec.	1.29	1.23	-	1.35	1.30	1.23	-	1.38	1.45	1.25	-	1.68	1.33	1.12	-	1.58	1.13	0.92	-	1.38	1.29	1.25	-	1.33
Upper sec.	1.16	1.10	-	1.22	1.16	1.09	-	1.24	1.02	0.86	-	1.20	0.99	0.82	-	1.20	1.18	0.96	-	1.46	1.14	1.10	-	1.18

§ Estimate based on 49 cases.

**Table 4:** Results from the Cox’s regression model: HRs of the association between education and cause specific mortality of residents in Rome, stratified by age group at inclusion and adjusted for birthplace. Females, 2001–2012.

Education (ref: post-secondary+)	Malignant neoplasms				Circulatory system				Respiratory system				Digestive system				Injury and poisoning				Total			
	HRs	95% CIs			HRs	95% CIs			HRs	95% CIs			HRs	95% CIs			HRs	95% CIs			HRs	95% CIs		
*All ages (30–74 yrs*.*)*																								
None	1.13	1.06	-	1.21	1.85	1.70	-	2.01	1.73	1.38	-	2.17	1.98	1.59	-	2.46	1.52	1.20	-	1.94	1.53	1.46	-	1.59
Primary	1.07	1.02	-	1.12	1.46	1.36	-	1.57	1.47	1.21	-	1.77	1.45	1.20	-	1.75	1.07	0.88	-	1.30	1.25	1.21	-	1.30
Lower sec.	1.10	1.04	-	1.15	1.29	1.20	-	1.39	1.38	1.13	-	1.69	1.38	1.14	-	1.68	1.18	0.96	-	1.44	1.20	1.15	-	1.24
Upper sec.	1.05	1.00	-	1.11	1.08	0.99	-	1.16	1.14	0.92	-	1.40	0.97	0.79	-	1.19	1.17	0.96	-	1.42	1.07	1.03	-	1.11
*30–44 yrs*.																								
None	2.68	1.64	-	4.37	12.31	6.11	-	24.82	60.59^#^	13.54	-	271.13	21.41^¶^	5.35	-	85.65	2.00	0.27	-	14.77	6.53	4.96	-	8.60
Primary	1.36	1.04	-	1.79	4.97	3.04	-	8.13	8.86	0.01	-	1.96	6.63	2.22	-	19.81	1.92	0.78	-	4.71	2.23	1.84	-	2.70
Lower sec.	1.31	1.12	-	1.52	2.86	1.97	-	4.17	2.72	0.14	-	0.73	5.30	2.23	-	12.58	2.10	1.30	-	3.40	1.74	1.54	-	1.97
Upper sec.	1.14	0.99	-	1.31	1.54	1.06	-	2.23	2.47	0.15	-	0.72	1.35	0.54	-	3.41	1.92	1.23	-	3.01	1.26	1.12	-	1.41
*45–59 yrs*.																								
None	1.35	1.12	-	1.64	4.31	3.20	-	5.81	5.02	2.48	-	10.16	4.08	2.16	-	7.71	2.14	1.03	-	4.45	2.26	1.98	-	2.57
Primary	1.15	1.05	-	1.25	2.34	1.92	-	2.85	1.95	1.17	-	3.26	1.44	0.93	-	2.22	0.71	0.45	-	1.12	1.36	1.26	-	1.46
Lower sec.	1.11	1.02	-	1.21	1.91	1.57	-	2.32	1.92	1.17	-	3.17	1.23	0.80	-	1.87	0.96	0.65	-	1.43	1.23	1.15	-	1.32
Upper sec.	1.08	1.00	-	1.18	1.24	1.01	-	1.52	1.02	0.59	-	1.75	1.09	0.72	-	1.67	1.03	0.70	-	1.50	1.10	1.03	-	1.18
*60–74 yrs*.																								
None	1.05	0.97	-	1.14	1.62	1.48	-	1.77	1.51	1.19	-	1.92	1.71	1.34	-	2.18	1.34	1.01	-	1.78	1.38	1.32	-	1.45
Primary	1.00	0.94	-	1.07	1.29	1.20	-	1.40	1.33	1.08	-	1.64	1.31	1.06	-	1.63	0.99	0.77	-	1.26	1.16	1.11	-	1.21
Lower sec.	1.04	0.98	-	1.12	1.13	1.04	-	1.23	1.24	0.99	-	1.55	1.24	0.99	-	1.56	1.04	0.80	-	1.35	1.11	1.06	-	1.16
Upper sec.	1.01	0.94	-	1.09	1.02	0.93	-	1.11	1.12	0.89	-	1.41	0.91	0.71	-	1.17	1.01	0.77	-	1.32	1.03	0.98	-	1.08

# Estimate based on 36 cases.

¶ Estimate based on 71 cases.

#### IPW estimates

All observations contributed to P estimates which ranged between 0.655 and 0.951 (SD 0.037). The strongest predictors of loss to follow-up due to emigration were being divorced compared to unmarried (OR 1.39, 95%CIs 1.35–1.43) and male gender (OR 1.26, 95%CIs 1.24–1.27), while the association with the educational background was at most 1.21 (95%CIs 1.19–1.23) compared with the uneducated people. Being unemployed, young, or born in Rome was positively associated with emigration to a lesser extent. Weighted estimates were similar to unweighted estimates as well as estimates resulting using stabilized weights.

## Discussion

The findings of this study based on the cohort of Italian residents in Rome in 2001, aged 30–74 years, and followed-up between 2001 and 2012, provide further evidence of an inverse association between educational level and overall mortality: the lower the education, the higher the risk to die. Assuming a causal association between education and mortality risk, the proportion of mortality in the entire population due to education less than the upper secondary level (population attributable fraction, PAF) is 0.13 (calculated using the gender-birthplace-adjusted HR in the Levin’s formula). A study analysing data from Turin found similar values of PAF in 2000 (0.1 females, 0.3 males) [26]. The association varied across strata of demographic characteristics, indicating the subgroup of the youngest women born in Rome as those being at the highest risk of differentials in mortality by educational level.

The decreasing vulnerability to low education according to increasing age, like the age-as-leveller hypothesis [27], may be expected as at older ages there may be many risk factors attenuating a possible effect of education on mortality. In addition, this may suggest the presence of additional risk factors correlated with deprivation among those young cohorts who did not reach standards of education in more recent years. Lower vulnerability to SEP observed at older ages may also reflect a selective mortality of unprivileged and more vulnerable individuals at younger ages [28].

We observed gender differences in mortality, with men being more vulnerable to the negative effect of low education on overall mortality. Gender differences in health are known: although women report more illness than men, they live longer than their counterparts as they are less affected by life-threatening diseases and their mortality rates are lower [29, 30]. A larger effect of education on men’s mortality rather than on women’s has been reported in a study by Ross et al. [31] and the authors invoke the role of education in the reduction of risky behaviours related to mortality for specific causes (violence, lung cancer, respiratory and liver disease) as an explanation.

Lower vulnerability to low education observed among people not born in Rome may reflect a different meaning of education as a proxy of socioeconomic circumstances according to place of birth: among those not born in Rome, education may be less relevant than other risk factors for mortality that may instead negatively confound the associations; for example, a protective effect of air quality in early life (less pollution in non urban areas) may be more evenly distributed across socioeconomic strata of people born in small towns or villages compared to people born in Rome, which is a large urban area with high levels of air pollution [32].

Also the analysis of cause-specific mortality indicated an inverse association with education, in particular for mortality related to respiratory, digestive or circulatory system, and again the youngest seemed more vulnerable to the effect of low education. Explanations for these results include smoking behaviour and alcohol use being more frequent among less educated or disadvantaged people [33, 34, 35].

The attenuated differentials observed among older women for cancer may be in part related to mortality for breast cancer, higher among high SEP women, and due to typical reproductive risks factors (late age at first delivery, not breastfeeding), before the spread of opportunistic or population screening programmes; or to initiation of cigarette use first among cohort of high educated women [36].

The inverse association observed between education and mortality for injuries and poisoning can reflect the effect of unsafe behaviours destructive to health (drug use, alcohol abuse, violence), typically more prevalent among less educated people, in particularly among men, that may influence mortality directly [31, 37]. This is in line with a study conducted in Italy on mortality between 1999 and 2007, which showed a pattern of increased mortality for external causes, in this case only for men [38].

Moreover, we appreciated lower vulnerability to low education among women compared to men for specific causes of mortality, in particular for respiratory and digestive systems, in accordance with the results reported by Ross et al [31].

### Strengths and limitations

This is the first study based on a large database of residents in Rome, the biggest Italian city, with a follow-up across a long time period of 12 years. It provides additional evidence on inequalities in mortality in the Mediterranean area using a longitudinal approach, showing interesting patterns of cause-specific mortality in relation to gender, age, and birthplace. In addition, the availability of information on individual educational level derived from the census guarantees the validity of the exposure measurement.

Different limitations should be considered for the interpretation of the results.

Loss to follow-up due to emigration may have introduced selection bias: if emigrants differed significantly from the remaining population in educational background, on the assumption that more educated people were more likely to emigrate, and that health status also was positively correlated with emigration risk, informative censoring may exist. In this case, the observed health advantage of education may be understated due to emigration of healthy educated persons. However, the educational background was not the strongest predictor of loss to follow-up. Moreover, the distribution of people by education according to emigration did not show marked heterogeneity: the proportion of people without education was 1.9% for people lost to follow-up vs. 2.8% for people not lost to follow-up, and 18% in the group with the highest level of education for both lost and not lost to follow-up. For this reason, assuming the absence of unmeasured confounding and of informative censoring, we decided to use the IPW approach in the regression models in order to handle missing information on outcome due to loss to follow-up due to emigration (12%) and we observed similar weighted HRs compared to unweighted estimates.

We found a slight deviation from the PHA which may suggest the presence of an interaction between education and time. Such deviation is easy to find in large databases and when we looked at differences from stratified analyses, according to time periods, we did not found relevant variations between estimates of HRs.

We did not use competitive risk analysis when we analysed cause-specific mortality. This aspect should be considered and caution adopted when interpreting associations in single causes. For example, differentials in mortality for diseases resulting fatal later in life (e.g. cardiovascular) compared to other (e.g. cancer), may appear as they are because of the selection effect of premature mortality.

Health selection should be also evaluated as a possible explanation of the association between mortality and SEP: specifically, ill people may be at higher risk of not completing qualified study courses than healthy people, thus introducing a reverse causality component in the results.

Although we cannot exclude other source of confounding besides age, gender, and birthplace, as well as factors related to the economic crisis and recession occurred during the study period, our results are consistent with those of other studies focusing on SEP and mortality conducted in Italy [38] as well as in other European countries [39].

### Conclusions

This is the first study based on a population cohort enrolled in the biggest Italian city, i.e. Rome, using a longitudinal approach to analyse differentials of overall and cause-specific mortality by educational level. The results provide further evidence from the Mediterranean area, and confirm the persistence of inequalities in mortality by education; they may contribute to national and cross-country comparisons in Europe, in order to understand the mechanisms generating socioeconomic inequalities in mortality.

Our findings call for the need to identify and promote appropriate interventions in the area of health, social, and environmental policies targeted to the disadvantaged population, which may act as remedial factors to reduce the observed differentials in mortality.

## Supporting Information

S1 Table Frequency distribution of the study population by educational level stratified by age group at inclusion, gender, and birthplace. Age 30–74 years, 2001.
(DOC)

S2 Table Frequency distribution of the study population by educational level stratified by age group at inclusion and cause of death. Age 30–74 years, males, 2001.
(DOC)

S3 Table Frequency distribution of the study population by educational level stratified by age group at inclusion and cause of death. Age 30–74 years, females, 2001.
(DOC)
