# Supplementary material for: Education and Mortality in the Rome Longitudinal Study
Source: PLoS One. 2015 Sep 16;10(9):e0137576. doi: 10.1371/journal.pone.0137576 (PMC4572712; doi:10.1371/journal.pone.0137576)
Supplement: S1 Table — Age 30–74 years, 2001. (DOC) [file pone.0137576.s001.doc]

**Table S1 - Frequency distribution of the study population by educational level stratified by age group at inclusion, gender, and birthplace. Age 30-74 years, 2001.**

| Educational level | **Born in Rome** | | |  | **Born elsewhere** | | |
| --- | --- | --- | --- | --- | --- | --- | --- |
|  | Males |  | Females |  | Males |  | Females |
|  | *30-44 yrs.* | | | | | | |
| None | 922 |  | 802 |  | 416 |  | 451 |
| Primary | 4,128 |  | 4,691 |  | 2,084 |  | 3,126 |
| Lower secondary | 52,436 |  | 45,022 |  | 16,827 |  | 16,210 |
| Upper secondary | 82,336 |  | 90,098 |  | 24,497 |  | 29,624 |
| Post-secondary+ | 32,346 |  | 36,082 |  | 15,761 |  | 20,927 |
|  | *45-59 yrs.* | | | | | | |
| None | 700 |  | 1,036 |  | 1,049 |  | 2,696 |
| Primary | 11,282 |  | 16,701 |  | 15,470 |  | 28,666 |
| Lower secondary | 28,970 |  | 32,796 |  | 27,987 |  | 30,870 |
| Upper secondary | 39,446 |  | 39,790 |  | 31,117 |  | 34,066 |
| Post-secondary+ | 20,967 |  | 18,394 |  | 20,650 |  | 20,768 |
|  | *60-74 yrs.* | | | | | | |
| None | 1,783 |  | 3,723 |  | 4,585 |  | 16,214 |
| Primary | 20,635 |  | 29,000 |  | 37,920 |  | 55,915 |
| Lower secondary | 20,761 |  | 23,365 |  | 24,055 |  | 23,502 |
| Upper secondary | 16,256 |  | 18,110 |  | 19,274 |  | 20,656 |
| Post-secondary+ | 10,428 |  | 7,020 |  | 16,933 |  | 11,425 |
