# Supplementary material for: Education and Mortality in the Rome Longitudinal Study
Source: PLoS One. 2015 Sep 16;10(9):e0137576. doi: 10.1371/journal.pone.0137576 (PMC4572712; doi:10.1371/journal.pone.0137576)
Supplement: S2 Table — Age 30–74 years, males, 2001. (DOC) [file pone.0137576.s002.doc]

**Supplemental table 2 - Frequency distribution of the study population by educational level stratified by age group at inclusion and cause of death. Age 30-74 years, males, 2001.**

| Educational level | Malignant neoplasms |  | Circulatory system |  | Respiratory system |  | Digestive system |  | Injury and poisoning |
| --- | --- | --- | --- | --- | --- | --- | --- | --- | --- |
|  |  |  |  |
|  | *30-44 yrs.* | | | | | | | | |
| None | 12 |  | 19 |  | 3 |  | 4 |  | 4 |
| Primary | 47 |  | 63 |  | 6 |  | 25 |  | 41 |
| Lower secondary | 442 |  | 400 |  | 27 |  | 108 |  | 268 |
| Upper secondary | 463 |  | 318 |  | 10 |  | 65 |  | 225 |
| Post-secondary+ | 158 |  | 94 |  | 3 |  | 11 |  | 90 |
|  | *45-59 yrs.* | | | | | | | | |
| None | 101 |  | 60 |  | 18 |  | 19 |  | 8 |
| Primary | 1,299 |  | 698 |  | 78 |  | 122 |  | 102 |
| Lower secondary | 1,907 |  | 1,143 |  | 85 |  | 176 |  | 164 |
| Upper secondary | 1,868 |  | 965 |  | 67 |  | 152 |  | 162 |
| Post-secondary+ | 902 |  | 475 |  | 35 |  | 49 |  | 77 |
|  | *60-74 yrs.* | | | | | | | | |
| None | 942 |  | 775 |  | 167 |  | 112 |  | 71 |
| Primary | 7,443 |  | 5,335 |  | 967 |  | 627 |  | 439 |
| Lower secondary | 4,901 |  | 3,487 |  | 566 |  | 409 |  | 258 |
| Upper secondary | 3,489 |  | 2,455 |  | 314 |  | 240 |  | 214 |
| Post-secondary+ | 2,410 |  | 1,729 |  | 254 |  | 196 |  | 149 |
