# Supplementary material for: Education and Mortality in the Rome Longitudinal Study
Source: PLoS One. 2015 Sep 16;10(9):e0137576. doi: 10.1371/journal.pone.0137576 (PMC4572712; doi:10.1371/journal.pone.0137576)
Supplement: S3 Table — Age 30–74 years, females, 2001. (DOC) [file pone.0137576.s003.doc]

**Supplemental table 3 - Frequency distribution of the study population by educational level stratified by age group at inclusion and cause of death. Age 30-74 years, females, 2001**

| Educational level | Malignant neoplasms |  | Circulatory system |  | Respiratory system |  | Digestive system |  | Injury and poisoning |
| --- | --- | --- | --- | --- | --- | --- | --- | --- | --- |
|  |  |  |  |
|  | *30-44 yrs.* | | | | | | | | |
| None | 17 |  | 10 |  | 4 |  | 3 |  | 1 |
| Primary | 66 |  | 29 |  | 4 |  | 7 |  | 6 |
| Lower secondary | 411 |  | 117 |  | 9 |  | 37 |  | 54 |
| Upper secondary | 685 |  | 121 |  | 16 |  | 18 |  | 99 |
| Post-secondary+ | 276 |  | 36 |  | 3 |  | 6 |  | 24 |
|  | *45-59 yrs.* | | | | | | | | |
| None | 123 |  | 66 |  | 13 |  | 14 |  | 9 |
| Primary | 1,263 |  | 428 |  | 58 |  | 60 |  | 36 |
| Lower secondary | 1,599 |  | 445 |  | 68 |  | 67 |  | 64 |
| Upper secondary | 1,702 |  | 311 |  | 38 |  | 66 |  | 77 |
| Post-secondary+ | 827 |  | 131 |  | 20 |  | 32 |  | 40 |
|  | *60-74 yrs.* | | | | | | | | |
| None | 1,355 |  | 1,465 |  | 190 |  | 202 |  | 130 |
| Primary | 5,414 |  | 4,575 |  | 668 |  | 617 |  | 375 |
| Lower secondary | 3,054 |  | 2,027 |  | 322 |  | 301 |  | 202 |
| Upper secondary | 2,447 |  | 1,522 |  | 240 |  | 183 |  | 162 |
| Post-secondary+ | 1,148 |  | 722 |  | 103 |  | 97 |  | 78 |
